# YAP/TAZ-mediated activation of serine metabolism and methylation regulation is critical for LKB1-deficient breast cancer progression

**DOI:** 10.1042/BSR20171072

**Published:** 2017-10-24

**Authors:** Qi Wu, Juanjuan Li, Si Sun, Xinyue Chen, Hanpu Zhang, Bei Li, Shengrong Sun

**Affiliations:** 1 Department of Breast and Thyroid Surgery, Renmin Hospital of Wuhan University, Wuhan, Hubei, P. R. China; 2 Department of Clinical Laboratory, Renmin Hospital of Wuhan University, Wuhan, Hubei, P. R. China

**Keywords:** breast cancer, histone methylation, LKB1, serine biosynthesis, YAP/TAZ

## Abstract

The crucial interplay between metabolic remodeling and the epigenetics could contribute to promote cancer progression. A remarkable association within interaction, LKB1 has been reported, suggesting that the expression of key enzymes involving *de novo* serine synthesis and DNA methyltransferases like DNMT1 and DNMT3A increase LKB1-deficiency cells. However, the complex interactional link between metabolic remodeling and the epigenetics is still unclear. Hence, we focus on the relationship between YAP/TAZ and serine metabolism to control methylation of DNA or histone in breast cancer with LKB1 deficiency. We hypothesize that YAP/TAZ may have the function to activate key enzymes involving serine metabolism like PSPH and up-regulate the amino acid transporters to supply sources of serine synthesis through activation of C-MYC with TEAD1. Further, we speculate that YAP/TAZ in dependent of FOS may promote DNMT1 and subsequently mediate DNMT1–G9A complex involving serine metabolism and the methylation of DNA and histone. We hope that our study will stimulate further studies and a new targeted therapy and early medical intervention for YAP/TAZ could be a useful option for breast cancer cases complicated with LKB1 deficiency.

## Introduction

Recent evidence suggests that the existence of important interplay between metabolic remodeling and the epigenetics such as histone methylation and acetylation can serve as a programmed switch in cancer cell states. Most chromatin-modifying enzymes require substrates or cofactors that are intermediates of cell metabolism, so changes in nutrient availability and utilization can influence epigenetic regulation [1,2]. Whether this interaction generally links to some oncogenic mutations and molecular drivers remains unclear, and this question has implications for understanding tumorigenesis and progression.

LKB1 controls a wide range of cellular functions that include metabolism, proliferation, and cell shape [3], and is mutationally inactivated in a range of sporadic cancers including breast cancer [4,5]. Cancers with low expression of LKB1 tend to exhibit poorer survival and different therapeutic sensitivity from cancers without these [6,7]. LKB1 directly activates AMP-activated protein kinase (AMPK) [8], which is a central regulator of cellular metabolism and energy homeostasis in mammalian tissues [9]. Recently, it demonstrates that significant up-regulation of the DNA methyltransferases DNMT1 and DNMT3A is found in LKB1-deficiency cells [10]. Therefore, the connection between metabolic remodeling and epigenetic regulation is thought to be a driver of tumorigenesis after LKB1 loss.

Serine consumption ranks second only to that of glutamine among the amino acids [11]. Serine can be either obtained from the diet or synthesized *de novo* from 3-phosphoglycerate (3-PG), an intermediate of glycolysis. Meanwhile, increased serine synthesis [12,13] and up-regulated serine transporter (SLC1A4) [14] has been identified in breast cancer tissues. Cancer cells with LKB1 loss increase the expression of phosphoserine aminotransferase 1 (PSAT1), phosphoserine phosphatase (PSPH) and serine hydroxylmethyltransferase (SHMT1/2) involving *de novo* serine synthesis pathway (SSP) in breast cancer [10]. In addition, the amplification of phosphoglycerate dehydrogenase (PHGDH), the first enzyme of the SSP and catalyze the conversion of 3-PG to 3-phosphohydroxypyruvate (3-PH), also occurs in some breast cancers. Cancer cells that support one-carbon unit demand by up-regulation of serine synthesis can do so via increased SSP genes expression. Moreover, a key regulator of SSP gene is ATF4 [15], a member of the basic region leucine zipper (bZIP) transcription factor family that can regulate gene transcription by forming a homodimer or heterodimer with other bZIP transcription factors and respond to stresses, such as amino acid deprivation [16]. ATF4 regulated by mechanistic target of rapamycin complex I (mTORC1) activation directly binds and activates the promoters of PHGDH, PSAT1, and SHMT2 [17]. Simultaneously, activated TORC1 promotes one-carbon metabolism for nucleotide synthesis by independently inducing ATF4-mediated SSP genes expression [18]. Besides, cells with LKB1 knockdown showed reduced activity of AMPK and subsequent activation of mTORC1 [10]. Therefore, the effects of serine metabolism on epigenetic regulation may be modulated by LKB1/mTORC1/ATF4 pathway.

Glutamine plays a predominant role in serine synthetic process, which provides nitrogen into a transamination reaction and produces α-ketoglutarate (α-KG) catalyzed by PSAT1. It has been shown that silencing LKB1 is sufficient to promote glutaminolysis and increase glutamine metabolism to fuel cell growth and lipid biosynthesis, which is mediated by the transcription factor HIF-1α that displays increased protein stabilization under normoxia when LKB1 is deleted [19]. Meanwhile, Yes-associated protein-1 (YAP1) directly enhances glutamine synthetase (GLUL) expression and activity, elevating steady-state levels of glutamine and enhancing the relative enrichment of nitrogen [20]. In addition, it demonstrates that YAP1 directly enhances GLUL expression and activity, and up-regulated expression of SLC38A1 and SLC7A5, main glutamine transporters [20–22]. Together, we speculate that YAP1 may increase glutamine level and enrich nitrogen to elevate synthesizing level of serine by enhancing GLUL expression and activity, elevating glutamine uptake, and enhancing the relative enrichment of nitrogen when LKB1 is silenced.

YAP and transcriptional co-activator with PDZ-binding motif (TAZ) are the major downstream effectors of the Hippo pathway, which was recently found to be regulated by metabolic pathways such as aerobic glycolysis [23]. Interestingly, YAP is activated and have significant implications in LKB1-deficiency human malignancies, mechanism of which depends on MARK/Scribble and is dependent on AMPK or mTORC1 [24]. Besides, ATF4 promotes the stabilization of the large tumor suppressor 1 (LATS1) under oxidative stress that inactivates YAP by phosphorylation [25] and specifically binds to the YAP promoter in HepG2 cells to enhance the transcriptional level of YAP [26]. Meanwhile, these reports indicate that YAP/TAZ may mediate up-regulation of key enzymes in SSP for one-carbon metabolism and tumor growth. As for serine metabolism, there is also evidence showing that TAZ S89A induces expression of the serine biosynthesis pathway (PHGDH, PASAT1, and PSPH) in C_2_C_12_ cells [27]. In addition, serine can be mainly synthesized from glucose and glycine and there is an evidence that YAP1 was found to positively regulate C-MYC and glucose transport-1 (GLUT1) mRNA levels in complex with TEAD1 [28] and it reports that activation of C-MYC also leads to elevate glutathione (GSH) production and drives PSPH to promote serine biosynthesis [29]. Thus, YAP/TAZ may be activated in LKB1-deficiency human malignancies depending on ATF4 up-regulation and increase serine synthesis through up-regulating key enzymes and supplying synthetic sources such as glucose.

**Figure 1:**
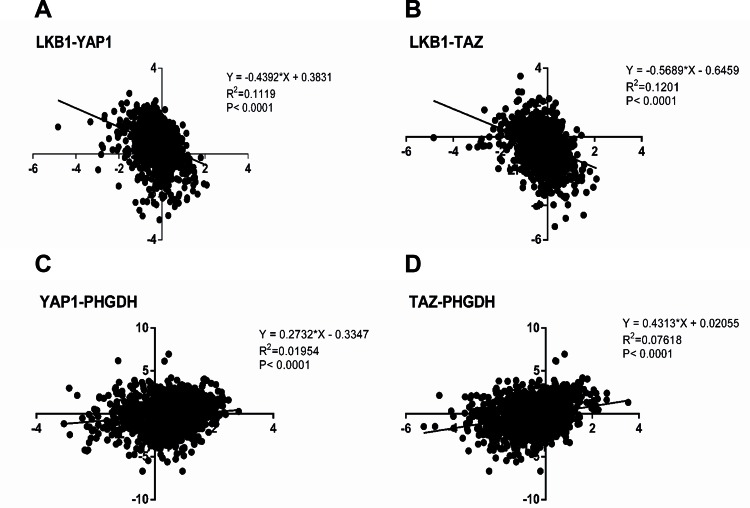
Correlation analysis for TAZ or YAP1 mRNA expression and LKB1 mRNA levels as well as TAZ or YAP1 mRNA expression and PHGDH mRNA levels in breast cancer tissues (**A**) After annotating the gene microarrays according to the TCGA 2012, we identify the expression levels of YAP1 were negative correlated with LKB1 level (*P*<0.0001). (**B**) The expression of TAZ and LKB1 in breast cancer tissues includes negative correlation (*P*<0.0001). (**C**) There is a positive correlation between the levels of YAP1 and the expression of PHGDH in breast cancer (*P*<0.0001). (**D**) The data from TCGA breast cancer suggest a positive correlation in TAZ expression compared with PHGDH level.

We also analyzed the correlation between TAZ or YAP1 mRNA expression and LKB1 mRNA levels as well as the relevance between TAZ or YAP1 mRNA expression and PHGDH mRNA levels in the breast cancer dataset from The Cancer Genome Atlas (TCGA 2012). In accordance with the observations, we find that the expression of TAZ or YAP1 and LKB1 are negatively correlated (*P*<0.0001), but there are positive correlations between TAZ or YAP1 expression and PHGDH level (*P*<0.0001) (Figure 1).

Methyltransferases and demethylases have a central role in regulation of transcription by controlling the methylation state of histone and DNA. S-adenosylmethionine (SAM) as the methyl group donor primarily derives from serine-driven one-carbon metabolism [30]. It recently identifies a G9A-dependent epigenetic mechanism for transcriptional activation of the serine pathway in cancer [31]. G9A, also known as EHMT2 and KMT1C, is a H3K9 methyltransferase that has a primary role in catalyzing H3K9me1 and H3K9me2 in euchromatin [32], with H3K9me1 being associated with active chromatin and H3K9me2 being a repressive mark [33,34]. Moreover, higher G9A expression significantly increases serine and glycine biosynthesis in the cell depending on up-regulation of ATF4 [31]. Multiple histone lysine demethylases (KDMs) catalyze the removal of methyl groups at H3K9: KDM3B can remove all methyl groups (me 1–3); KDM4 only me2 and me3; and KDM3A and KDM7A-B only me1 and me2 [34,33]. Thus, the KDM4C requires ATF4 for the transcriptionally control of amino acid including serine biosynthesis and transport by removing the repressive marks H3K9me2 and H3K9me3 at their loci [35]. As for DNMTs, it confirms that DNMT1 interacts with ATF4, FOS, and FOSB and is activated [36]. Meanwhile, DNMT1 as the primary loading factor is shown to directly bind G9a both *in vivo* and *in vitro* and to colocalize with dimethylated H3K9 (H3K9me2) at replication foci [37]. And YAP1 converges on the transcription factor FOS and activates a transcriptional program [38], which may include DNMT1. Simultaneously, Yorkie (YAP homologs) can activate transcription by recruiting NcoA6, a subunit of Trithorax-related (Trr)-methyltransferase complexes, which links the control of cell proliferation by Hippo signaling to epigenetic modification [39]. It has also demonstrated that Set7, a SET-domain-containing lysine methyltransferase, could monomethylate Yap at K494 and maintain cytoplasmic localization [40]. Therefore, there is a complex interrelationship between methylation regulation of DNA or nonhistone proteins and function of YAP, which should be further established.

## Consequences of the hypothesis and discussion

**Figure 2:**
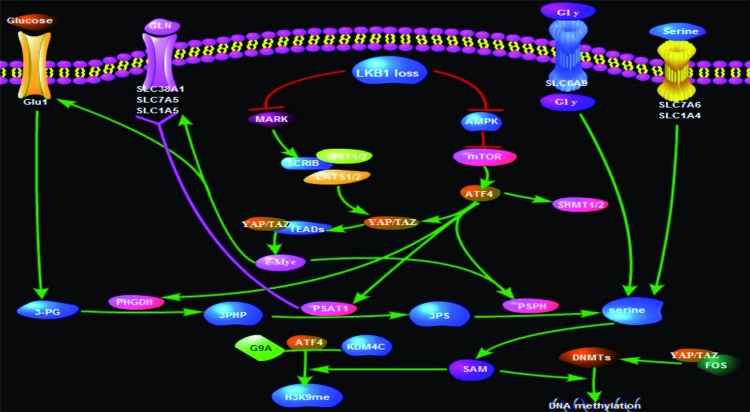
Working model for YAP/TAZ-mediated activation of serine metabolism and methylation regulation in LKB1-deficient breast cancer progression Working model for YAP/TAZ-mediated activation of serine metabolism and methylation regulation in LKB1-deficient breast cancer progression.

In summary, for breast cancer cells with LKB1-deficient, YAP/TAZ is up-regulated while key enzymes involving serine metabolism and methylation-regulation are activated. Further, high expression of TAZ S89A could directly promote the expression of key enzymes involving serine metabolism, and YAP/TAZ modulates up-regulation of the amino acid transporters (SLC38A1, SLC7A5, and SLC3A2) and GLUT1 to supply sources of serine synthesis through activation of C-MYC with TEAD1. Subsequently, ATF4 that is reported to modulate the serine metabolism could specifically bind to the Yap promoter to enhance the transcriptional level of YAP. In addition, the methylation of DNA and histone involving in this process is not unambiguously elucidated, and some transmethylases like G9A and DNMTs and demethyltransferases like KDM4C might participate in the regulation. Hence, we hypothesize that YAP/TAZ may have the function to link serine metabolism to methylation in a subset of breast cancer patients with LKB1 deficient (Figure 2). Further, we speculate that YAP/TAZ in dependent or independent manner of ATF4 promote key enzymes and transporters involving serine metabolism and the methylation of DNA and histone.

Our hypothesis points to the role of YAP/TAZ in the regulation of serine metabolism and histone methylation in a subset of breast cancer patients with LKB1 deficient. The presence of YAP/TAZ overexpression in breast cancer cases complicated with LKB1 deficient could worsen survival and contribute to cancer progression. If our hypothesis is confirmed by further studies, a new targeted therapy and early medical intervention for YAP/TAZ could be a useful option for a subgroup of breast cancer cases complicated with LKB1 deficient, which may have important clinical and health implication.

## Abbreviations

α-KG: α-ketoglutarate
3-PG: 3-phosphoglycerate
3-PH: 3-phosphohydroxypyruvate
AMPK: AMP-activated protein kinase
bZIP: basic region leucine zipper
GLUL: glutamine synthetase
GLUT1: glucose transport-1
GSH: glutathione
LATS1: large tumor suppressor 1
PHGDH: phosphoglycerate dehydrogenase
PSAT1: phosphoserine aminotransferase 1
PSPH: phosphoserine phosphatase
SHMT1/2: serine hydroxylmethyltransferase
SSP: serine synthesis pathway
